# Chia seeds (*Salvia hispanica* L.): A therapeutic weapon in metabolic disorders

**DOI:** 10.1002/fsn3.3035

**Published:** 2022-12-15

**Authors:** Waseem Khalid, Muhammad Sajid Arshad, Afifa Aziz, Muhammad Abdul Rahim, Tahira Batool Qaisrani, Fareed Afzal, Anwar Ali, Muhammad Modassar Ali Nawaz Ranjha, Muhammad Zubair Khalid, Faqir Muhammad Anjum

**Affiliations:** ^1^ Department of Food Science Government College University Faisalabad Faisalabad Pakistan; ^2^ Department of Agricultural Engineering and Technology Ghazi University Dera Ghazi Khan Pakistan; ^3^ Department of Epidemiology and Health Statistics, Xiangya School of Public Health Central South University Hunan China; ^4^ Institute of Food Science and Nutrition University of Sargodha Sargodha Pakistan; ^5^ Islamic Food and Nutrition Council of America IFANCA Halal Apex, Private Limited Faisalabad Pakistan

**Keywords:** cancer, cardiovascular disease, chia seeds, functional components, GI tract

## Abstract

The growth of functional components containing agricultural foods is enhancing because these components aid the human body against different chronic diseases. Currently, chia seeds basically belong to the mint family and are edible seeds of *Salvia hispanica*. These seeds are composed of different functional components including fiber, polyphenols, antioxidants, omega‐3 fatty acid vitamins, minerals, and peptides. Besides, these seeds are also a good source of vegetable protein, unsaturated fat, carbohydrates, and ash. Chia seed components are helpful in cardiovascular disease (CVD) by reducing blood pressure, platelet aggregation, cholesterol, and oxidation. In GI‐tract‐related diseases like diabetes and constipation, chia fiber reduces the blood glucose level and provides bulk to stool. However, antioxidants and polyphenols are protected beta cells of the pancreas from inflammation. These components are protected from the cell damage of the different body parts, which can provide help in different types of cancer including breast, colorectal, liver, and pancreatic. Conclusively, some pervious studies approved that chia seed components are played important role in chronic diseases.

## Graphical abstract

Conclusively, chia seeds components are played important role in chronic diseases that approved by different previous studies.

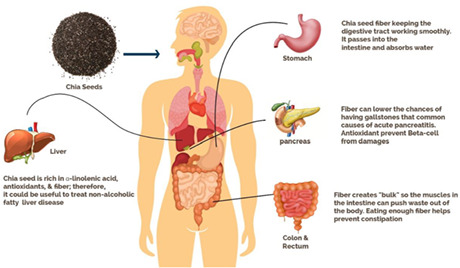

## 1 INTRODUCTION

In the recent times, the science of functional foods is a combination of science and collective needs. It consists of nutrition, food science and medicine that maintains a balance between food and drugs. The word functional food was invented first in Japan at 1984 (Shimizu, 2003). Functional foods has been defined by the National Academy of Sciences Food and Nutrition Board in the USA; “Any reconstituted food or food component that can be good for health more than its conventional nutritive value” (Martirosyan & Pisarski, 2017). Functional foods are commonly divided into two major categories, such as conventional and genetically modified food prodcuts (Rahim et al., 2022). (Di Renzo et al., 2020). Furthermore, genetically modified foods are prepared with the addition of vitamins, minerals, probiotic microflora, and fiber. Fortified juices, fortified dairy products are the best examples of these products and easy available in the local markets (Gupta, 2014). These foods contain phytochemicals that can help to protect from cell damage, heart disease, cancer and keep balance against diabetes.

## 2 CHIA SEED AND ITS NUTRITIONAL COMPOSITION

Chia seeds are the consumable seeds of *Salvia hispanica*. It basically belongs to the mint family (*Lamiaceae*). Chia seeds are egg shaped and gray with black and white stains, bearing a width of about 2 millimeters (0.08 inches). Chia (*Salvia hispanica* L.) belongs to the class Labiatae which is originated from Mexico, Northern, and Guatemala (Madaan et al., 2020). Chia (*Salvia hispanica*) is an annual blossoming plant. These seeds were ingested as food. Commonly Chia seeds are cultivated as a blend of both white and black seeds. Now a days, black chia seeds are most of the seeds cultivated that are identified by spiral and striped coloring. White chia seeds are present in a very low amount and a bit greater than black seeds. To enhance the yield of white seeds more as output, peasants segregated the white seeds prior to sowing to have more white seeds plants. Gardeners employ a color segregation technique to grow homogenous seeds for seeding, and they may also use the same method after collecting the seeds. It is exhibited by testing that in the past white and black chia seeds were cultured separately, but with time, the production of white chia seeds decreased and intermixed with high‐yielding black seeds (Ayerza, 2010). Chia seed is chemically composed of vegetable protein, lipids, carbohydrates, and dietary fiber which are listed in Table 1. Recent outcomes of different investigations show that chia seeds have a great nutritional profile and huge health‐boosting characteristics (Grancieri et al., 2019). Chia seed is attributed to have useful results on the betterment of the blood lipid profile, by their ability in lowering blood pressure, blood glucose, and antimicrobial and immuno‐boosting reactions (Weber et al., 1991).

**TABLE 1:** Nutritional composition of chia seeds.

Parameter	USDA (2018)	da Silva et al. (2017)	Jin et al. (2012)	Basuny et al. (2021)
Protein	16.5 g/100 g	18.9 g/100 g	24.5 g/100 g	28.33%
Fat	30.7	31.2	40.2	37.53
Carbohydrate	42.1	–	26.9	–
Ash	4.8	–	4.77	3.90
Dietary fiber	34.4	35.3	30.2	35.45
Energy	480 Kcal	–	562 Kcal	–

## 3 FUNCTIONAL COMPOSITION OF CHIA SEED

### 3.1 Fiber

Fiber is an insoluble carbohydrate for our body (Mudgil & Barak, 2013). It is present in edible plants like fruits, vegetables, grains, and legumes (Khan et al., 2007). Fiber is of two types including soluble and insoluble. Cellulose, a type of fiber that present in grains, fruits, vegetables, nuts, and beans, that cannot digest in our body (Gidley & Yakubov, 2019). It passes through the body without digestion and reducing constipation or helping in maintaining a healthy GI tract. It also aid in and rinsing the cholesterol and cancer‐causing chemicals from our body (Holscher, 2017). Chia seeds carry 40% fiber that brings them to the top of the list in providing more fiber (Reyes‐Caudillo et al., 2008). Soaked seeds carry dietary fiber. Its present in the form of a gel that helps the stool in its movement. Fiber improves the motion of stool. Soluble fiber rises the volume and bulkiness of feces that regulates blood sugar level, body weight, and cholesterol level, and the health of the colon also acts as antiaging (Grancieri et al., 2019; Rahim et al., 2021). Wet chia seeds are sticky in nature, due to mucilage and digestible fiber. These are useful in controlling blood sugar after eating, and provide satiety. Earlier studies claimed that soluble fiber can upgrade the consistency of digestive material so connected with health benefits to humans (Table 2). Artificial tools were designed to see the assimilation of mucilage in chia seeds. Three amounts of mucilage were used 3, 5, and 8 g/kg to assess the differences in the digestion process. Mucilage had a tremendous capacity to hold water (Lazaro et al., 2018).

**TABLE 2:** Different functional components of chia seeds and their potential health benefits.

Functional component	Present in part	Helpful in diseases	References
Fiber	Bran	Diabetes, CVD, and Constipation	Fernandes et al. (2021), Liu et al. (2002), Oliveira‐Alves et al. (2017)
Antioxidants	Whole seed	Cancer and Diabetes	Alwosais et al. (2021), da Silva Marineli et al. (2014), Hajhashemi et al. (2010)
Polyphenols	Whole seed	Cancer and Alzheimer's disease	Oliveira‐Alves et al. (2017), Shabbir et al. (2021)
Vitamins (E)	Whole seed	Cancer	EFSA (2009), Yang et al. (2012)
Minerals	Bran	Reduce BP	Ghafoor et al. (2018), Taneja and Mandal (2007)
Omega‐3 Fatty acid	Mostly in endosperm	Reduce bad cholesterol and TG	Basuny et al. (2021), Pizzini et al. (2017), Porras‐Loaiza et al. (2014)

### 3.2 Antioxidants

Antioxidants are substances that slow down oxidation. Oxidation, a process that creates free radicals in a series that can also harm cells. The phytochemicals including antioxidants, phenolic compound and ascorbic acid that function to stop oxidation process (Aversa et al., 2016). Foods including vegetables and fruits, are superior sources of photochemicals thats known to be good for health (Ignat et al., 2011). Chia seed is a feasible source of phytochemicals having chlorogenic acid, caffeic acid, myricetin, quercetin, and kaempferol, which are assumed to have a defensive reaction for the heart and liver, and also have age‐defying and anticancerous properties (Melo et al., 2019; Ullah et al., 2016). Small‐size chia seeds have immune‐boosting effects by having microchemicals and omega‐3 fatty acids. It lowers the sensitivity and swelling reactions through maintenance (Lokhande et al., 2019). A dessert of chia seeds can make to enhancing the concumption of chia seeds. The second radiant zone of chia seeds is their rich amount of phytonutrients (Table 2). Hence, scientists accepted during the discussion that taking additives of these chemicals will effect one’s life productively (da Silva Marineli et al., 2014). Another study showed that, these chemicals have a defensive reaction against the creation of harmful substances for cells (Pellegrini et al., 2018).

### 3.3 Phenolic compounds

Phenols are a group of chemical substances having one or more hydroxyl groups (OH) linked to an aromatic hydrocarbon (Nomura et al., 2002). These are categorized on the basis of a number of phenolic groups present in a compound. Phenolic components are a major category of plant derivatives and are separated as phenolic acids and polyphenols. These are present in combination with mono‐ and polysaccharides, having one or more phenolic groups, which may turn out as ester or methyl esters (Kumar & Goel, 2019). Phenolic acids, flavonoids, and tannins are termed principal nutritional phenolic components in comparison to other categories of phenolic compounds (Stalikas, 2007). A powerful and productive connection between phenolic components and photochemical capability of fruits and vegetables is proved by several studies (Rajashekar et al., 2009). The plant‐based mechanism is of vital importance for the limitation of lipid oxidation in living tissues because when this is added to the diet of individuals, it improves the way of living and also lowered the chance of happening disorders. The more fruits and vegetables in food can play a role in slowing the age‐defying process, reduction in swelling and oxidative stress causing long‐lasting disorders including cardiovascular diseases, arteriosclerosis, cancer, diabetes, cataract, cognitive function, and neurological disorders (Minatel et al., 2017). Phenolic and polyphenolic components alone or in relation to vitamins, like carotenoids, vitamin E, and vitamin C, are reducers that safeguard the special tissues of humans from oxidative stress. Fruits and vegetables have plenty of antioxidants, most commonly polyphenols. Caffeic and rosmarinic acids (Capitani et al., 2012; Reyes‐Caudillo et al., 2008) are phenolic substances and previously recognized in chia‐based things; they have a key function in the inhibition and controlling many congenital diseases just like epilepsy (Martínez‐Cruz & Paredes‐López, 2014). Oliveira‐Alves et al. (2017) showed that phenolic components from profit‐oriented samples of chia seeds, flour, dietary fiber, and oil were separated using different technologies. The mixture of raw and compounded were examined, major substances present were phenolic and caffeic acid, and danshensu and their secondary metabolites like rosmarinic and salvianolic acids. Total phenolic compounds were more in dissolved solutions. These outcomes provided updated knowledge about chia seeds and their phenolic profile, which was mainly phytochemicals and fibers for stopping oxidative stress and disorders caused by it (Scapin et al., 2016). Table 2 shows the health benefits of chia seeds' phenolic compounds.

### 3.4 Omega‐3 fatty acid

Omega‐3 fatty acids, also known as omega‐3 oils, ω‐3 fatty acids, or n‐3 fatty acids, are unsaturated fats distinguished characterized by the existence of a double bond, three atoms apart from the side methyl group in its chemical composition (Innis, 2008). Mainly these are key components in the digestion of animal fats and also play a vital function in human food and physical functioning (Cholewski et al., 2018). These are divided into three types including α‐linolenic acid (ALA), present in plants, and eicosapentaenoic acid (EPA) and docosahexaenoic acid (DHA) both are widely present in fish oils (Dyall, 2015). Simple sources of herbal oils having ALA are walnut, wholesome seeds, chia seed, clary sage seed oil, flaxseed oil, Sacha Inchi oil, Echium oil, and hemp oil (Simopoulos, 2002). Vertebrates are not able to produce ALA; they attain ALA from plants' food only. However, they utilize ALA, when accessible, to synthesize EPA and DHA, form extra double bonds on its terminal carbon chain (unsaturation), and expand them (expansion). Specifically, ALA (18 carbons chain and 3 double bonds) is manipulated to create EPA (20 carbons and 5 double bonds), then that is work to form DHA (22 carbon atoms and 6 double bonds) (Adeyemi et al., 2020). The branching capability of omega‐3 fatty acids by ALA can be weakened in oldness. Omega‐3 fatty acids revealed to oxygen are more at risk to be spoil and oxidize (Veena et al., 2017). One of the distinctive features of chia seeds is their high levels of omega‐3 fatty acids good for cardiac health. Omega‐3 alpha‐linolenic acids (ALA) are about 75% and 20% omega‐6 fatty acids present in chia seeds. It is an effortless way to boost your mental health by just consuming chia seeds. Previous study showed that eating more omega‐3 than omega‐6 will lower infections and swelling of your body. A little ratio of omega‐3 fatty acid is linked with a decreased chance of many long‐lasting diseases like heart problem, cancer, inflammation, and premature deaths (Saini & Keum, 2018).

### 3.5 Peptides

Peptides are formed by linking 2 and 50 amino acids together, with peptide bonds. Ten or fifteen amino acids are joined to form oligopeptides and may also dipeptides, tripeptides, and tetrapeptides. Amino acids are the basic unit of proteins, but proteins have more amino acids in them (Gentilucci et al., 2010). Peptides are lower in molecular weight so are more smoothly consumed than proteins. The different kinds of peptides are ribosomal peptides that normally performing the hormonal function. These peptides are generated by the cells until “propeptides” or “proproteins” are shortened before departing the cell. They are liberated into the blood to execute signaling roles (Gu et al., 2011). Peptides are cellular entities consisting of amino acids that are responsible for regulating functional systems in our body. Multidimensional protein identification tecnologies are used for multiple purpose like their relevancy in medicines, peptide hormones, amyloid composition, self‐building compositions, colloidal gels, and peptide associates as well as lipo‐peptides and polymer–peptide conjoined. Plant proteins may achieve or substitute animal proteins as a best source of necessary essential amino acids, (Montoya‐Rodríguez et al., 2015). Chia seeds have conatined around 19% protein. Its consider to be a good source of proteins. Chia seeds have elevated levels of proteins and necessary amino acids, therefore these are a favorable source of functional peptides. The structure and useful outcomes of protein and peptides in chia seeds (*S. hispanica* L.), have possible influences on human health. The details were applied to explore the peptides' functional capability. Of total proteins, 20 are classified in chia seeds. These are a consistent part of plant's basal metabolism (Gómez‐Favela et al., 2017). Examination of amino acid order exhibited that peptides with functional capability have dipeptidyl peptidase‐IV inhibitors, angiotensin‐converting enzyme inhibitors, and antioxidant ability. The beneficial chia seed effects are linked to humans by having the antioxidant ability, antihypertensive, antihyperglycemic, and anticholesterolemic reactions. These connections are may be linked with chia protein and peptide structures (Grancieri et al., 2019).

### 3.6 Vitamins

Vitamin is a natural substance that is an indispensable micronutrient that an organism requires in low amounts for the appropriate activity of its cellular functions (Zempleni et al., 2013). All necessary vitamins are not be produced inside the body. These vitamins take from different foods. Vitamins are one of many organic nutrients, which are required in low amounts, for the maintenance of health and growth at an advanced level of animal life (McDowell, 2008). Vitamins are different in a number of ways from other functionally significant molecules like proteins, carbohydrates, and lipids. These molecules are also essential for the regular functioning of organisms, most of them are manufactured in sufficient amounts by animals. Vitamins are not formed by the body in enough amounts to fulfill the demands of the body therefore it is necessarily to gained through diet or some artificial means (Halver, 2003). Vitamins also contradict the other biomolecules. Therefore, these are comparatively less required in proportions to accomplish their roles. Commonly they are regulators and activators in nature that accelerating and managing all the integral chemical reactions in the body (Panzeca et al., 2006).

The seeds contain high amounts of B vitamins including niacin (883 mg/100 g), folic acid (49 mg/100 g), thiamine (0.62 mg/100 g), and riboflavin (0.17 mg/100 g) (Olivos‐Lugo et al., 2010). Kulczyński et al. (2019) found that chia seeds contain vitamin E as tocopherols: α‐tocopherol (8 mg/kg of lipids), γ‐tocopherol (422 mg/kg of lipids), and δ‐tocopherol (15 mg/kg of lipids). Chia seeds are rich source of several important minerals. Chia seeds are good for boosting the metabolism. Apart from this, their high levels of omega‐6 acids in absorbing fat‐soluble vitamins A, D, E, and K (Muñoz et al., 2013).

### 3.7 Minerals

Minerals are earthly components that are necessary for natural bodywork, functioning, and development. Our body requires several minerals. These are known as essential minerals (Miller, 2017). These are categorized into substantial elements (macronutrients) and trace elements (micronutrients). Both of these have identical significance, but microelements are desired in minor quantity than the major elements (Turan et al., 2003).

Chia seeds are an extraordinary origin of numerous fundamental minerals but an imperfect source of vitamins. These are abundant in manganese, phosphorus, copper, selenium, iron, magnesium, and calcium. Similar to vitamins, minerals keep you healthy and also aid in the growth and development of the body. The body utilizes minerals to carry out various activities, from the construction of powerful bones to the traveling of nerve impulses. However, different minerals are used to form hormones or for the normalization of a heartbeat (Bailey et al., 2011). Chia seeds exceptionally have a high amount of numerous necessary minerals but are not a good source of vitamins. These have high levels of magnesium, calcium, manganese, phosphorus, copper, selenium, and iron (da Silva et al., 2017). Whole meals and grains are abundant in manganese, which is needed for ingestion, digestion, maturation, and progression (Aschner & Dorman, 2006). Normally present in foods that are high in proteins (chia seed). Phosphorus provides health to bones and helps sustain tissues (Takeda et al., 2012). As a mineral that is frequently deficient in recent foodstuffs. Copper is crucial for cardiac health (Allen & Klevay, 1994). A major phytonutrient (selenium), is responsible for different systems in our body (Rayman, 2012). Magnesium is mostly insufficient in Western food. it performs a vital role in various bodily mechanisms (Blaszczyk & Duda‐Chodak, 2013). The surplus mineral in our body like calcium is critical for bones, muscles, and nerves (Emkey & Emkey, 2012). The functional role of different minerals is listed in Table 2.

## 4 A FUNCTIONAL PERSPECTIVE OF CHIA SEEDS IN VARIOUS DISEASES

### 4.1 Reduce the risk of cardiovascular disease (CVD)

**FIGURE 1:**
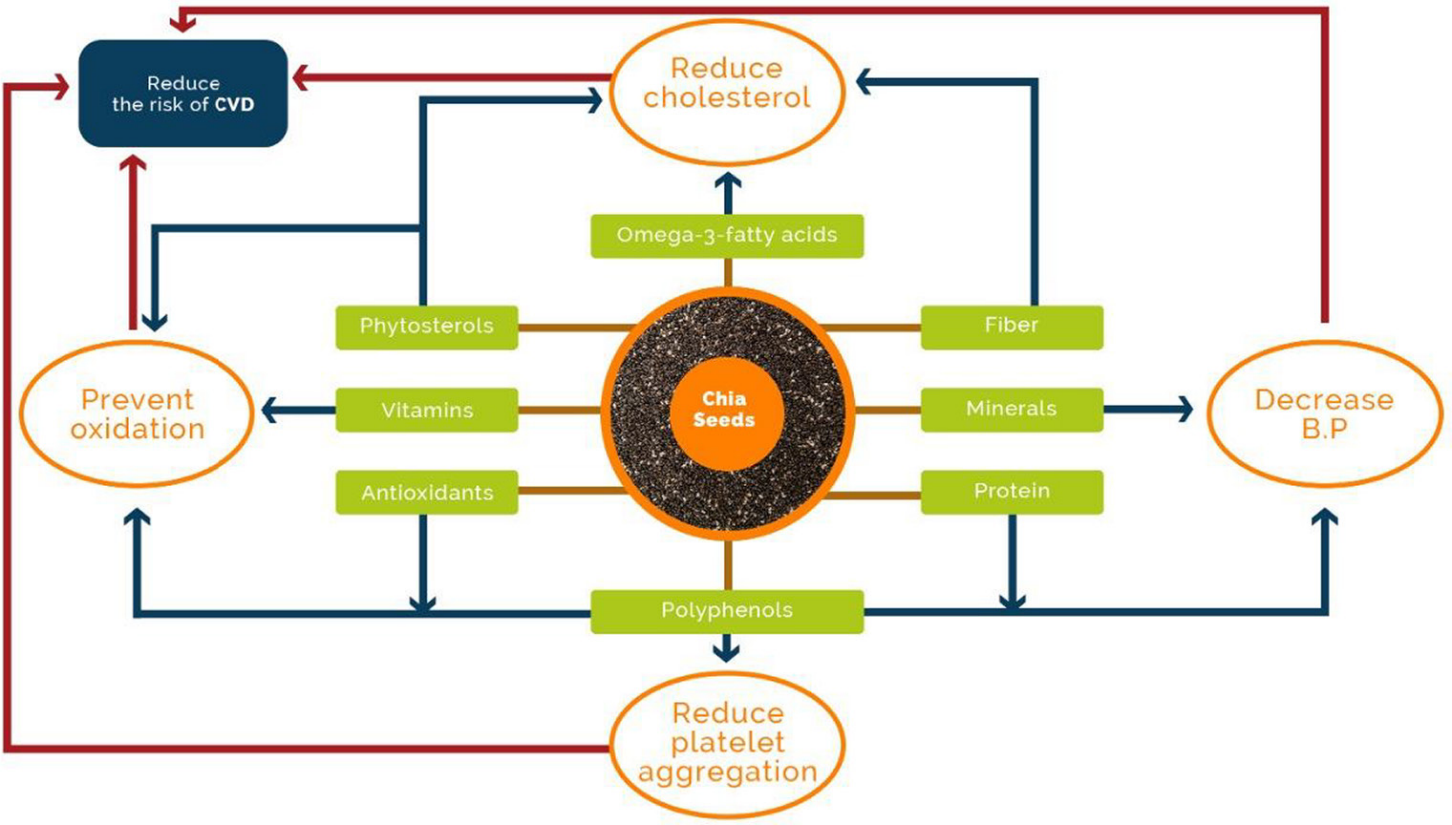
Chia seeds' functional components that aid in reducing the risk of cardiovascular disease.

Cardiovascular disease (CVD) is a universal phrase for the state disturbing the heart or blood vessels. CVD, also known as heart and cardiac arrest, is equivalent to the state that influences your heart or blood flow. It is typically related to the accumulation of fat on the inside of blood vessels and a greater chance of blood clumps (Nangia et al., 2016). It may also be interrelated with the destruction of the other parts of the body, for example, the heart, brain, eyes, and kidney. This covers hypertension, metabolic syndrome, muscle inactivity, diabetes mellitus, and other disorders. Coronary artery illness comprises situations that shrink or choke the arteries (coronary infarction) to your heart muscle (Kurian & Cardarelli, 2007). The chief physiological threat components of cardiac infarction and failure are occurred due to more intake of unbalanced diet patterns, lack of exercise, smoking, and unfavorable use of liquor. The consequences of physiological hazards are high blood pressure, high blood glucose level, high lipid profile fleshiness, and chubbiness (American Diabetes Association, 2005). Chia seeds’ contain omega‐3 fatty acids and antioxidants that can be used as a functional component aid in reducing the risk of CVD (Figure 1).

Minimizing salt and alcohol utilization, and adding fruits, vegetables, and exercise in one’s life are indicated lowering the chance of cardiovascular ailment (Eilat‐Adar et al., 2013). Moreover, medicinal treatment of hyperglycemia, high blood pressure, and lipids content are also essential in lowering and controlling the happening of this disorder. Health plans that generate favorable conditions for building healthy way‐outs, inexpensive, and convenient are necessary for stimulating people to maintain and encourage healthy habits (Psaty et al., 1999).

Dietis a key factor in heart disease. Dietary‐based likelihoods involve overweight, hypertension, unchecked high blood glucose, and taking more animal fats in food. Diet rich in plant fats, soluble fibers, and organic food can significantly lower the origination of coronary diseases. Furthermore, low‐standard dietary foods are more in processed grains, salt, sugars, and saturated fats from animals; are limited in fresh fruits, vegetables, nuts, legumes; and are low in unprocessed grains (Bechthold et al., 2019). They mostly contain refined food products usually prepared and fresh and contain few whole foods and newly made products (Popkin et al., 2012; WHO, 2010). Considering the high energy and supplement thickness of seeds, the overall part of seeds in the prerural eating regimen is justifiable. Seeds are likewise especially significant in human nourishment because of their interesting piece of organically dynamic mixtures. It is significant that somewhat recently, a lot of logical proof depends on the advantageous impacts of the expanded utilization of plant seeds and inferred items on different well‐being results (mainly CVD, diabetes mellitus type 2 (DM2), and intermediate markers) (Ros & Hu, 2013). The seed is a small embryonic plant encapsulated in a covering known as seed coat, which is the product of mature ovules of flowering plants after pollination and reproduction. The seed is composed of a complex external matrix and a germ rich in biologically active minerals, vitamins, and phytochemicals, which can protect the DNA of the plant from oxidative stress and promote the perpetuation of the species (Raven et al., 2005). Minimizing salt and alcohol utilization, adding fruits, vegetables and exercise in your life indicated in lowering the chance of CVD (Eila‐t Adar et al., 2013). Moreover, medicinal treatment of hyperglycemia, high blood pressure and lipids content are also essential in lowering and controlling the happening of this disorder. Health plans that generate favorable conditions for building healthy wayouts, inexpensive and convenience are necessary for stimulating people to maintain and encourage healthy habits (Psaty et al., 1999).

#### 4.1.1 Reduced triglyceride fat

Triglycerides (TG) are esters derived from glycerol and three fatty acids. Fatty acids enriched oils are the fundamental parts of the body and plant fats in people and different vertebrates. Fatty substances are a sort of fat (lipid) in the blood. Fatty oils are put away in your fat cells. Afterward, chemicals discharge fatty substances between suppers to give energy (Lestari et al., 2009). High fatty substances can cause arteriosclerosis, or thickening of the course dividers (arteriosclerosis), expanding the danger of stroke, cardiovascular failure, and coronary illness. High fatty substances can likewise cause intense aggravation of the pancreas (pancreatitis) (Zhang et al., 2019).

Chia seeds contain essential substances including high fiber, protein, and omega‐3 fatty acids, they can decrease the development of TG. Its advantages have been demonstrated in certain examinations, however, the outcomes are uncertain (Enes et al., 2020). Numerous study on observation and experimental have shown that chia seeds can reduced the certain danger factors, including fatty oils, aggravation, insulin opposition, and stomach fat. They can likewise expand “great” HDL cholesterol. They are an outstanding source of omega‐3 fatty acids, rich in antioxidants, and provide fiber, iron, and calcium (Ayerza Jr & Coates, 2007). The results demonstrated that chia seed oil supplementation changed the lipid profile in the liver and adipose tissue, decreased leptin, triglycerides, and hepatic cholesterol (Citelli et al., 2016).

#### 4.1.2 Improve High‐density lipoprotein (HDL)

Cholesterol is a waxy substance found in all cells and has numerous valuable capacities, including making a difference in human cell form. It is transmitted through the blood bound to the protein. These proteins are called lipoproteins (Cook, 2015). High‐density lipoprotein (HDL) cholesterol is designated “acceptable” cholesterol since it helps eliminate different types of cholesterol from the blood. Significant degrees of HDL are related to a lower hazard of coronary illness. Therefore, HDL cholesterol is frequently called “acceptable” cholesterol. HDL assimilates the overabundance of cholesterol from the blood and afterward returns it to the liver, where it is separated and taken out from the body (Cardenas et al., 2008).

Contrasted and immersed fats and vegetable oils abundant in polyunsaturated fats diminish the proportion of TC: HDL‐C (complete cholesterol: HDL‐C) and the rate of coronary illness by adding omega‐3 unsaturated fats (α‐linolenic corrosive) to vegetable oils. It is fundamental for the counteraction of coronary illness (Flock & Kris‐Etherton, 2012). The impact of supplanting immersed fat with carbs on the danger of coronary illness seems to rely upon the nature of the sugars. Forthcoming investigations have reliably shown the unfriendly impacts of transfats on coronary illness. The impact of plant‐based monounsaturated fats needs more exploration; additional virgin olive oil seems to decrease CVD (Plat et al., 2019).

Chia seeds are wealthy in fiber and contain omega‐3 unsaturated fattty acids, because of their high substance of soluble fiber, chia seeds can assimilate up to 10–12 times their weight in water, turn thick, and swell in the stomach (Alfredo et al., 2009). In principle, this should build satiety, moderate food ingestion, and assist you with decreasing your calorie consumption. Soluble Fiber can also feed beneficial bacteria in the intestinal tract, which is absolutely essential for maintaining good flora nutrition and intestinal health (Alfredo et al., 2009). Chia seeds are rich in omega‐3 fatty acids. It contain essential fatty acids like that alpha‐linolenic acid (ALA) and oelic acids. After comsumption, theses essential fatty acids are converted in the human body to the eicosapentaenoic acid (EPA) and docosahexaenoic acid (DHA). (Jin et al., 2012). Omega‐3 unsaturated fattty acids can increased the level of HDL cholesterol, which is the “acceptable” cholesterol that secures against coronary failures and strokes. Studies have shown that chia seeds (particularly, ground chia seeds) can build blood levels of ALA and EPA (Nieman et al., 2012). These long‐chain omega‐3 polyunsaturated fatty acids (PUFAs) have useful impacts in the counteraction of cardiovascular and provocative illnesses. The first unsaturated fat ALA (18: 3, n‐3) found in vegetable oils, for example, linseed oil or rapeseed oil, is utilized by human life forms as a wellspring of energy and, to some extent, as a forerunner of metabolites, yet the level of change is by all accounts inconsistent (Gerster, 1998). This examination researched the impact of the dietary admission of chia seeds (*Salvia hispanica* L.) abundant in alpha‐linolenic corrosive and fiber on dyslipidemia and insulin resistance (IR), which was caused by the intake of foods rich in sucrose (62.5%) (SRD). Chia seeds in the diet can reduce visceral fat in SRD rats. This study provides new data on the useful effects of chia seeds on lipid and glucose homeostasis in lipid‐ and IR‐positive experimental models (Chicco et al., 2008).

#### 4.1.3 Reduced the blood pressure

Circulatory strain is the pressing factor of the circulation system on the dividers of the veins. (McQueen et al., 1955). High blood pressure can damage the arteries, block them, and impede blood flow to the heart muscle. High blood pressure can cause blood vessels in the brain to become blocked or even break more easily. The increased workload of high blood pressure can cause the heart to expand and not supply blood to the body (Jordan et al., 2018).

Chia seeds contain quercetin, an antioxidant (da Silva Marineli et al., 2014), that can reduced the danger of different medical issues, including coronary illness and risk factors of heart diaseases. A few investigations have shown that chia seeds can altogether bring down the pulse rate in patients with hypertension, which is an incredible danger factor for coronary illness (Toscano et al., 2014).

### 4.2 Helpful in the gastrointestinal tract‐related diseases

The human digestive system comprises the gastrointestinal lot in addition to assistant stomach‐related organs. Absorption includes separating the food into more modest segments until it can be assimilated and consumed by the body. The digestive system is comprised of the gastrointestinal tract (likewise called the gastrointestinal parcel or stomach‐related lot), just as the liver, pancreas, and gallbladder (Hillman et al., 2017). The gastrointestinal tract is a series of hollow organs that are connected to each other from the mouth to the anus. The empty organs that make up the gastrointestinal tract are the mouth, throat, stomach, small digestive tract, internal organ, and rear end (Manson et al., 2008). The primary capacity of the gastrointestinal tract is to process and assimilate ingested supplements and discharge digested waste (Kim & Pritts, 2017). The form of intake of most nutrients is too complicated, indigestible, or insoluble, making digestion difficult or impossible. Various diet modifiers, including live edible microbes (probiotics) and consumable food fixings (e.g., prebiotics), and polyphenols, are the most described organically dynamic mixtures in the eating regimen and have been demonstrated to be advantageous (Forootan et al., 2018). Chia seeds are a likely wellspring of cancer prevention agents within the sight of chlorogenic corrosive, caffeic corrosive, myricetin, quercetin, and kaempferol. It is accepted to have heart and liver defensive against maturing and hostile to disease properties. It is likewise a significant wellspring of dietary fiber. It is wealthy in higher convergences of gainful unsaturated fatty acids, without gluten proteins, nutrients, minerals, and phenolic compounds, which are advantageous for the digestive system and to control diabetes (Ullah et al., 2016).

Polyunsaturated unsaturated fats (PUFAs) (counting omega‐3 and omega‐6 unsaturated fats) and phytochemicals assume a significant part as naturally dynamic mixtures in a solid eating regimen (Laparra & Sanz, 2010). The balance of PUFA components in food affects all aspects of immunity and metabolism (Laparra & Sanz, 2010). Furthermore, the interaction among PUFAs and segments of the intestinal verdure can likewise influenced their natural impacts. Chia seeds containing omega‐3 unsaturated fatty acids have been related to a large group of physiological capacities in the human body. Phytochemicals (nonnutritive plant compounds with natural movement) have stood out because of their likely impact as cell reinforcements, antiestrogens, mitigating drugs, immunomodulators, and anticancer medications (Avrelija & Walter, 2010; Laparra & Sanz, 2010). The intestinal vegetation can be for instance, change and impact the bioavailability and impacts of polyphenols (Laparra & Sanz, 2010). Phytochemicals and their metabolites can also inhibit pathogenic bacteria (Avrelija & Walter, 2010; Laparra & Sanz, 2010), and simultaneously animate the development of gainful microorganisms, assuming a part like that of prebiotics. Cooperations between practical food fixings, for example, prebiotics, probiotics, phytochemicals, and intestinal greenery, affect human well‐being (Laparra & Sanz, 2010).

**FIGURE 2:**
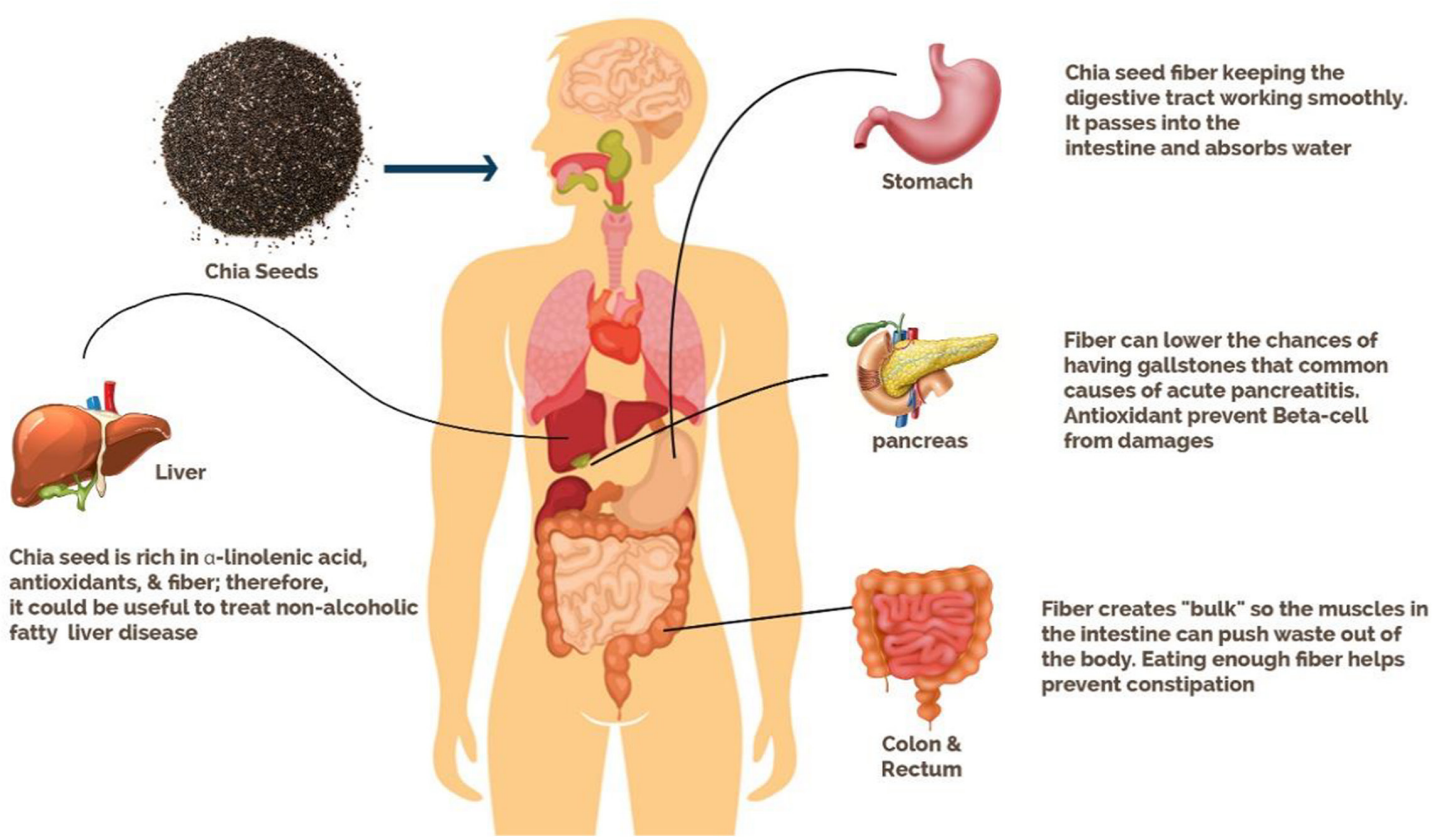
Functional role of chia seed components in the human gastrointestinal tract.

Intestinal epithelial cells (IEC) in the covering of the gastrointestinal tract make a hindrance between the outside and inner climate. A total intestinal hindrance forestalls tissue harm, microbe contamination, and infection improvement, in this manner keeping up intestinal well‐being and the general strength of the body. At the point when the capacity of the intestinal obstruction is upset, a bacterial movement can happened in the body. Our intestinal verdure assumes on a very basic level a significant part in well‐being, for instance, keeping up the uprightness of the intestinal hindrance, digestion, and directing the invulnerable framework. Any harm to the structure of the intestinal vegetation (otherwise called ailing health) can prompt different obsessive conditions. So, intestinal obstruction and intestinal verdure are two key factors that influence intestinal well‐being. The gastrointestinal tract is a perplexing climate presented to numerous dietary parts and harmonious microbes. Individuals are progressively aware that dietary ingredients can perform several beneficial functions in addition to basic nutrition, which has led to the improvement of the concept of purposeful foods. Many diet modifiers, comprising of live edible microbes (probiotics) and edible food ingredients (such as prebiotics) and polyphenols or symbiotics (combination of probiotics and prebiotics), are the best biologically active compounds in the diet. The role of the intestinal epithelium and intestinal flora in the defense response of the mucosa and the influence of certain functional components of food in the regulation of intestinal health including probiotics, prebiotics, and polyphenols (Wan et al., 2019). Figure 2 shows the functional role of chia seed components in the human gastrointestinal tract.

#### 4.2.1 Type 2 diabetes

Type 2 diabetes is a common disease that causes high levels of sugar (glucose) in the blood. It can cause symptoms such as thirst, the need to urinate a lot, and tiredness. It can also increase your risk of serious eye, heart, and nerve problems (DeFronzo et al., 2015). The dietary fiber contained in food varieties, particularly entire grains, has become a significant organic segment because of its potential medical advantages. An enormous number of studies have shown the impacts of fiber utilization as diminishing the danger of coronary illness, decreasing the danger of type 2 diabetes, and different sorts of malignant growth (Reyes‐Caudillo et al., 2008). Consuming dietary fiber is linked to increased satiety after a meal and reduced hunger afterward. According to data from the American Dietetic Association, dietary fiber has shown benefits for maintaining health and preventing chronic disease (USDA 2000). Chia seeds contain 34 to 40g of dietary fiber per 100 grams, which is equivalent to 100% of the recommended daily intake for adults; the fiber content of skimmed flour is 40%, of which 5–10% is soluble and mucilage (Mohd Ali et al., 2012). Therefore, chia seeds can be used to reduce the risk of cardiovascular diseases and diabetes.

Foods low in saturated fatty acids have become a daily chore and in recent years the demand for these products has increased in the developing and underdeveloping countries. The main reason for the change from saturated fat to unsaturated fat is increased the number of patients of cardiovascular disease, high blood pressure corpulence, diabetes, and other well‐being‐related infections in humans (Hansel et al., 2007). The previous study summarizes that the process of phenolic compound biosynthesis in plants, including the oxalic acid, pentose phosphate, and phenylpropane pathways. Plant phenolic mixtures can go about as cell reinforcements, underlying polymers (lignin), attractants (flavonoids and carotenoids), UV insurance specialists (flavonoids), flagging mixtures (salicylic corrosive and flavonoids), and protective response synthetics (tannins and phytotoxins). From the point of view of human physiology, phenolic compounds are fundamental for guard reactions, for example, against maturing, mitigating, cell reinforcement, and hostile to proliferative exercises. Subsequently, it is gainful to burn through food sources of plants beginning with a high substance of cell reinforcement compounds, which can reduced the rate of certain constant infections like diabetes, malignant growth, and cardiovascular illnesses by controlling oxidative pressure. Besides, berries and different natural products with low amylase and high glucosidase inhibitory exercises can be viewed as a dietary possibility to control the beginning phases of hyperglycemia related to type 2 diabetes (Lin et al., 2016).

Phenolic compounds, synthetic parts removed from plants, can be repressed the ingestion of amylase in the treatment of sugar assimilation (like diabetes). The phenolic compounds are particularly produced from chia seeds. Phenolic compounds, like phenolic acids and flavonoids, can advance well‐being by decreasing the danger of difficulties identified with metabolic conditions and type 2 diabetes (Sales et al., 2012).

#### 4.2.2 Constipation

Constipation is a condition in which reduce the bowel movements, and hard stools that are painfull to pass. The fluid piece of the stool is assimilated once more into the body, making the stool hard and dry. This makes it hard for stool to pass (Camilleri et al., 2017).

Chia seeds give insoluble dietary fiber, which keeps up completion longer, expands defecations, and forestalls obstruction. They additionally give solid fats, proteins, and cancer prevention agents that secure cells (Knez Hrnčič et al., 2020; Tan & Seow‐Choen, 2007). Control of solid discharges, dietary fiber expands the weight, size of stools, and mellows them. Stool and defecation are simpler to pass, diminishing the opportunity of blockage. On the off chance that one’s stool is free and watery, the dietary fiber can help cement the stool by engrossing dampness and expanding stool mass (Korczak et al., 2017). An eating routine wealthy in fiber can lessen the danger of hemorrhoids and colonic packs (diverticulosis). Studies have additionally tracked down that an eating regimen wealthy in fiber can reduced the risk of colorectal disease. A portion of the fiber is fermented in the colon due to the cannot be completely breakdown by human digestive enzymes. (Tang et al., 2020).

It is perceived that the consistency and gel‐shaping properties of solvent dietary fiber can hinder macronutrient retention, decrease postprandial glucose reaction, and advantageously influence certain blood lipids. Colonic maturation of normally accessible fiber‐rich food sources can be principally credited to dissolvable dietary fiber, and as far as weight guidelines, no distinction was seen between solvent and insoluble dietary fiber consumption. Nonetheless, in forthcoming partner contemplates, major insoluble dietary fiber grains and entire grains were utilized rather than insoluble dietary fiber, which is constantly connected with reduce the risk of diabetes, recommending that other obscure systems might be included (Cui & Roberts, 2009). The examinations have shown that taking dietary fiber can caused numerous unexpected metabolic impacts that have nothing to do with changes in body weight, including improving insulin affectability, controlling the discharge of certain intestinal chemicals, different metabolic, provocative responses identified with metabolic chemicals, effect of checking, and metabolic disorder. (Weickert & Pfeiffer, 2008).

### 4.3 Cancer

A term for a disease in which abnormal cell division is uncontrolled and invades close by tissues. Cancer growth cells can spread to different parts of the body through the blood and lymphatic framework. There are a few primary types of cancer. the most common type is malignancy that starts in the skin or in the tissues or tissues that line interior organs. Sarcoma is another type of cancer that starts in the bones, ligaments, fat, muscles, veins, or other connective or backing tissues. Leukemia is a kind of disease that starts in the blood‐shaping tissues (like the bone marrow) and leads to an overproduction of abnormal blood cells lymphoma, and various myeloma are malignancies that begin in cells of the resistant framework. Tumor of focal sensory system is a serious disease that starts in the tissue of the mind and spinal string and, when extreme or dangerous can have exceptionally high death rate (Shaikh et al., 2021; Steele, 2021). Symptoms and indications of cancer growth rely upon the particular sort and grade of disease. Although the overall manifestations and signs are not exceptionally clear, the accompanying conditions can be found in patients with various tumors: exhaustion, weight reduction, torment, changes in the skin, digestive system or changes in bladder work, strange dying, hack steady or changes in voice, fever, irregularities, or masses of tissue (Cleeland et al., 2003).

Omega‐3 fatty acids are additionally expected to give wholesome enhancements to malignant growth patients. Not at all like omega‐3 and omega‐6 fatty acids have calming impacts and do not advance angiogenesis (Caughey et al., 1996). Consequently, omega‐3 supplementation to patients gives the benefit of nourishment, particularly fat, without animating tumor development. In certain examinations (Colomer et al., 2007; van der Meij et al., 2011), omega‐3 supplementation may help to increase the weight of malignancy patients. Omega‐3 impacts improving cachexia in disease patients (Ries et al., 2012). Notwithstanding, the admission of omega‐3 fatty acids causes genuine antagonistic responses (Arends et al., 2017). Furthermore, antioxidants are very important in preventing cancer because they help to perevent free radical demage that have been associared with cancer cells development. Cancer prevention agents are synthetic compounds that block the movement of different synthetic compounds (called free radical) (Gupta et al., 2014). Research scientist have shown that exogenous cell reinforcements can help free extreme harm related to disease improvement (Poljsak & Milisav, 2018). Some proof recommends that higher calcium may reduce the risk of cancer (particularly, colorectal disease). T Chia seeds are wealthy in fiber, protein, magnesium, manganese, and calcium, which is a phenomenal wellspring of cell reinforcements (Grancieri et al., 2019). Chia seeds are a richest plant source of omega‐3 polyunsaturated fatty acids, which can forestall aggravation, improve psychological capacity, and lower cholesterol levels. Chia seeds are additionally rich in polyphenols got from caffeic acid, and these phenols are cell reinforcements that shield the body from free radicales, maturing, and malignancy. (De Falco et al., 2017).

Chia seeds and oil are rich source of tocopherols, phytosterols, and carotenoids (Álvarez‐Chávez et al., 2008), and polyphenolic compounds (principally made out of primary units of caffeic acid and flavonoids), myricetin, quercetin, and kaempferol. The presence of these compounds have ability to scavenge free radicals, to chelate ions particles, and to give hydrogen. Specifically, the B‐ring of flavonoids is required to explain the unsystematically movement of ROS and RNS, since hydrogen and electrons are moved to hydroxyl, peroxy acid, and peroxynitrite to balance them out, subsequently producing generally stable flavonoid (Cao et al., 1997). Table 3 presented the functional role of chia seeds against different types of cancer. Reinforcements of cell forreducing the risk of infections, including cancer and coronary illness, and give assurance against specific sicknesses.

**TABLE 3:** Therapeutic role of chia seeds to prevent different types of cancer.

Cancer Type	Common in Gender	Risk Factors	Name of Functional Components in Chia seeds	Reducing Mechanism	References
Breast Cancer	Female	Radiation exposure and obesity	Antioxidants and omega‐3 fatty acid	Omega‐3‐ atty acids reduce the TG and bad cholesterol levels that prevent the body from being overweight. Antioxidants inhibit the oxidation process that can aid to prevent cell damage.	Hu et al. (2021), James et al. (2015), Prathyusha et al. (2019)
Colorectal Cancer	Male	Inflammatory bowel disease and adenomatous polyps	Fiber and antioxidants	Fiber is not digestible in the body that inhibits constipation by softening the stool. Antioxidants aid in inhibiting inflammation of cells by providing the radical during oxidation.	Knez Hrnčič et al. (2020), Lukas (2010), Muñoz et al. (2012), Schmitt and Greten (2021)
Liver Cancer	Male	Chronic infection, diabetes, and nonalcoholic fatty liver disease	Phenolic compound, fiber, and omega‐3 fatty acid	Omega‐3 fatty acids and fiber can reduce the chances of saturated fat in the body that are suitable for a healthy liver. Phenolic compounds act as an antioxidant that can reduce inflammation.	Coates (2011), Fernández‐Palanca et al. (2021), Muñoz et al. (2012), Sun and Karin (2012)
Pancreatic Cancer	Male	Obesity, diet, and diabetes	Tocopherols, phytosterols, fiber, and polyphenols	Tocopherols and phytosterols reduce bad cholesterol and prevent obesity. Fiber is a nondigestible polysaccharide that reduces blood glucose and cholesterol levels.	Kulczyński et al. (2019), Yadav and Lowenfels (2013)

## 5 CONCLUSION

It is concluded that functional components of agricultural food such as chia seeds have therapeutic importance. Chia seeds contain more than one functional component. The importance of these components has the ability to suppress the risk of chronic diseases including GI‐tract‐related diseases, CVD, and various types of cancer. Fiber, omega‐3 fatty acid, protein, polyphenols, phytosterols, vitamins, and minerals reduce heart diseases by controlling bad cholesterol, hypertension, and platelet aggregation. In the GI tract, chia seed components reduce type 2 diabetes by improving the beta‐cell performance and reducing the blood glucose level. Moreover, chia seeds have rich fiber that provides bulk to stool, so these seeds can prevent constipation. However, antioxidants and phenolic part of these seeds improve oxidation and aid in reducing the risk of different types of cancer. In the future, Chia seed components may be used as an additive ingredient in different food products such as meat products and baking products to improve the nutrition value and shelf stability.

## 6 ACKNOWLEDGEMENTS

The authors are thankful to department of Food Science, Government College University Faisalabad for providing the free subscription of articles to complete this manuscript.
